# Comparison of CD4+/CD8+ Lymphocytic Subpopulations Pre- and Post-Antituberculosis Treatment in Patients with Diabetes and Tuberculosis

**DOI:** 10.3390/pathogens12091181

**Published:** 2023-09-20

**Authors:** Erick J. Rendón Ramírez, Adrián G. Rosas-Taraco, Berenice Soto-Monciváis, Perla R. Colunga-Pedraza, Rosario Salazar-Riojas, Nereida Méndez-Ramírez, Alma Yolanda Arce-Mendoza, Antonio Muñiz-Buenrostro, Jorge Llaca-Díaz, David Gomez-Almaguer, Adrián Rendón

**Affiliations:** 1 Servicio de Neumología y Medicina Crítica, Hospital Universitario “Dr. José Eleuterio González”, Universidad Autónoma de Nuevo León, Monterrey 64460, Mexico; 2 Departamento de Inmunología, Facultad de Medicina, Universidad Autónoma de Nuevo León, Monterrey 64460, Mexico; 3 Centro de Investigación, Prevención y Tratamiento de Infecciones Respiratorias (CIPTIR), Hospital Universitario “Dr. José Eleuterio González”, Universidad Autónoma de Nuevo León, Monterrey 64460, Mexico; 4 Servicio de Hematología, Hospital Universitario “Dr. José Eleuterio González”, Universidad Autónoma de Nuevo León, Monterrey 64460, Mexico; perla.colungapd@uanl.edu.mx (P.R.C.-P.);; 5 Departamento de Patología Clínica, Hospital Universitario “Dr. José Eleuterio González”, Universidad Autónoma de Nuevo León, Monterrey 64460, Mexico

**Keywords:** adaptive immunity, latent tuberculosis, lymphocytic subpopulations, QuantiFERON, diabetes, active tuberculosis

## Abstract

Is there a CD4+ and CD8+ immunity alteration in patients with pulmonary tuberculosis (TB) and diabetes (DM) that does not recover after antituberculosis treatment? This prospective comparative study evaluated CD4+ and CD8+ lymphocytic subpopulations and antituberculosis antibodies in patients with diabetes and tuberculosis (TB-DM), before and after antituberculosis treatment. CD4+ T cell counts were lower in patients with TB-DM compared to those with only TB or only DM, and these levels remained low even after two months of anti-TB treatment. Regarding the CD8+ T cell analysis, we identified higher blood values in the DM-only group, which may be explained by the high prevalence of latent tuberculosis (LTBI) in patients with DM. IgM antituberculosis antibodies levels were elevated in patients with only TB at baseline, and 2 months post-anti-TB treatment, IgG did not express any relevant alterations. Our results suggest an alteration in CD4+ immunity in patients with TB-DM that did not normalize after antituberculosis treatment.

## 1. Introduction

Tuberculosis (TB) and diabetes mellitus (DM) represent significant global health challenges, particularly in low-income countries. The coexistence of these two conditions, TB-DM, has drawn increasing attention due to its prevalence and complex interactions between the diseases [1,2]. TB is estimated to affect 10.6 million individuals worldwide annually, while DM has reached a global prevalence of 537 million, with projections anticipating a further rise by 2045 [3,4]. The Mexico and Texas border reported a DM prevalence of 25% among TB cases [5]. Notably, individuals with DM face a 3–4-fold increased risk of developing active TB, making the co-occurrence of these conditions a significant public health concern [6,7].

DM in patients with TB has been associated with impaired cellular immunity, altered lymphocyte subpopulations, and aberrant cytokine production. CD4+ T cells and their cytokine profile (IL-12, IFN-γ, TNF-α, IL-17, and IL-23) are the main immunity alterations, with IFN-γ and TNF-α being the most important ones [8,9,10,11]. On the other hand, CD8+ T cells have a protective role in immunity against TB; they can recognize infected cells and produce cytokines such as TNF and IFN-γ, lyse infected cells, and kill *Mycobacterium tuberculosis* (MTB), though not as effectively as CD4+ T cells. Therefore, CD8+ T cells also play a critical role in preventing the reactivation of latent tuberculosis infection (LTBI) [12].

The tuberculin skin test (TST) and QuantiFERON-TB Gold Plus (QFT-Plus) give an indirect qualitative measure of immune cellular function against TB. QFT-Plus, in addition, is useful in diagnosing LTBI in the general population, especially in DM, which is in agreement with the WHO’s recommendations as a method to identify and treat LTBI, avoiding TB reactivation [4].

Finally, regarding the humoral immunity, IgM and IgG antibodies against MTB are crucial to trigger the adaptive immune response. These antibodies have been of value in diagnosing lung TB in patients with negative smear in sputum, and their positivity indicates active TB [13,14].

The aforementioned immunological abnormalities have been implicated in the progression from LTBI to active disease in patients with DM. This increased risk has been associated with higher levels of glycosylated hemoglobin and lipid profile alterations [15].

Despite extensive research on immune dysfunction in patients with TB-DM, there remains a scarcity of data on the dynamics of immunological characteristics before and after antituberculosis treatment in this population [16,17]. This knowledge gap prompted us to carry out a prospective, comparative study to investigate the impact of antituberculosis treatment on adaptive immunity in patients with TB-DM. 

The main objective of our study focused on evaluating CD4+ and CD8+ T cell behavior before and two months after anti-TB treatment in patients with DM. In addition, our secondary objectives were the evaluation of anti-MTB antibodies in patients with TB-DM, as well as the cellular immunity evaluation with TST and QFT-Plus.

## 2. Materials and Methods

### 2.1. Study Population

We conducted an observational, comparative study from October 2018 to September 2022 at the University Hospital “Dr. José Eleuterio González”, which is a referral center for TB in Monterrey, Mexico. The Internal Institutional Review Board (IRB) approved the study with protocol code NM18-00007 to ensure compliance with ethical guidelines and patient confidentiality. We included four groups of participants: (1) healthy subjects, (2) patients with only DM, (3) patients with only pulmonary TB, and (4) patients with pulmonary TB-DM. Biochemical parameters and cellular/humoral immunity were measured at baseline, and a second measurement of the latter was taken after 2 months, only in the TB groups (only Tb and TB-DM). Eligible patients were adults (aged ≥18 years) of any gender, recently diagnosed with TB, with or without DM. We excluded patients with a diagnosis of human immunodeficiency virus (HIV) infection, use of corticosteroids, the presence of bacterial or fungal infection, immunosuppression due to chemotherapy or the use of biologics, active cancer of any kind, collagen, hematological diseases, pregnancy, or breastfeeding.

Active TB was defined through detecting acid-fast bacilli in Ziehl–Neelsen staining of sputum or bronchoalveolar lavage, positive culture, or positive mycobacterial PCR. DM was defined according to American Diabetes Association (AHA) criteria [18]. Several biochemical parameters were analyzed, including fasting glucose, lipid profile, urea, creatinine, transaminases, and HbA1c. Anthropometric measurements, including weight and height, were also collected. 

### 2.2. Lymphocytes Subpopulations

Complete peripheral blood was used to evaluate the lymphocyte subpopulations in healthy subjects and patients via flow cytometry. The blood sample (50 μL) assessed absolute cell counts using BD Multitest™ CD3/CD8/CD45/CD4, Becton Dickinson, USA and BD Trucount™ Tubes, Becton Dickinson, USA. Samples were incubated for 30 min at room temperature; erythrocytes were lysed using a LNW protocol. After staining, 2500 lymphocytes were acquired in a FACS Canto II flow cytometer, Becton Dickinson, San José California, USA, and were analyzed using FACS Canto software version 3.0, Becton Dickinson, San José, CA, USA.

### 2.3. Cellular Immunity

Cellular immunity was assessed through the tuberculin skin test (TST) and QuantiFERON-TB Gold Plus (QFT-Plus) at the time of inclusion.

### 2.4. Anti-MTB Antibodies

Indirect ELISA tests were carried out according to the patent No. 285260 called “Proceso de detección de tuberculosis”, developed in the Immunology Department of the Medical School in the UANL (Arce-Mendoza and Rosas-Taraco, 2011). First, 1 µg/well of the antigen diluted in acetate buffer pH 7.2–7.4 was placed in 96-well costar plates. The plates were incubated overnight at 4 °C; then, supernatants were discarded, and the plates were blocked with 200 µL of 5% diluted skim milk in phosphate buffer. Serums of controls and patients were used to evaluate the levels of anti-MTB protein antibodies [19]. Samples were diluted at 1:50 in 1% skim milk Peroxidase-conjugated anti-human IgM and IgG antibodies were diluted at 1:10,000. The plates were read in an iMark™ spectrophotometer (Bio-Rad, Tokyo, Japan) at λ = 490 nm.

### 2.5. Statistical Analysis

Data were analyzed using software SPSS v.20.0 and Graphpad prism. Descriptive statistics were used to describe demographic variables. We used the chi-square test for categorical variables and ANOVA (post hoc Tukey) or Kruskal–Wallis (post hoc Dunn) for quantitative variables among the different groups, according to their distribution. A *p*-value of <0.05 was considered statistically significant.

## 3. Results

### 3.1. Demographics Data and Metabolic Parameters

A total of 62 patients were included, divided into four groups: (1) healthy subjects, (2) patients with only DM, (3) patients with only TB, and (4) patients with TB-DM. The mean age in the TB-only group was 27 (18–84) years vs. 46 (22–71) years in the TB-DM group, *p* < 0.001 (Table 1).

**Figure 1:**
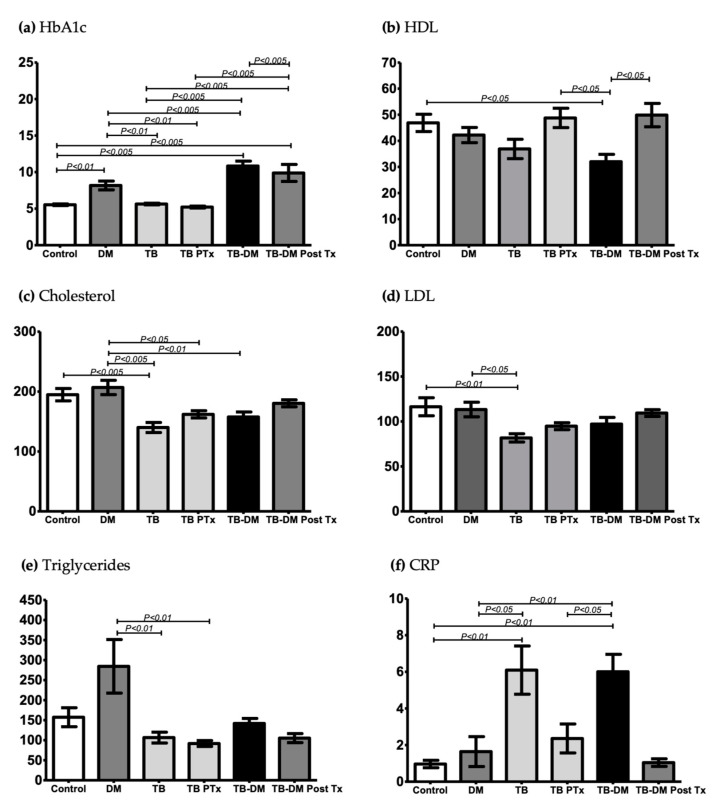
Plots showing the metabolic profile of controls and patients’ groups: diabetes mellitus (DM, *n* = 15), tuberculosis (TB, *n* = 17), tuberculosis post-treatment (TBPTx, *n* = 12), tuberculosis with diabetes mellitus (TB-DM, *n* = 15), and tuberculosis with diabetes mellitus post-treatment (TB-DM PostTx, *n* = 7). (**a**) HbA1c percentage; (**b**) HDL, mg/dL; (**c**) cholesterol, mg/dL; (**d**) LDL, mg/dL; (**e**) triglycerides, mg/dL; (**f**) C-reactive protein (CRP), mg/dL. ANOVA and Tukey’s multiple comparisons were used in the HbA1c, HDL, cholesterol, and LDL statistical analysis. The triglyceride and CRP statistical analysis used Kruskal–Wallis and Dunn’s multiple comparisons. The values are expressed as mean ± SD.

Hb1ac was 10.77 (±2.66 SD) in the TB-DM group vs. 8.17 (±2.2 SD) in the DM-only group, with *p* < 0.001. The comparison between other groups was also statistically significant (Table 1, Figure 1a). HDL cholesterol was 32 (±10.9 SD) mg/dL in TB-DM at baseline vs. 49.8 (±11.8 SD) mg/dL in TB-DM post-anti-TB treatment, with *p* < 0.05 (Figure 1b). Basal total cholesterol was 180 mg/dL (±15.3 SD) in patients with TB-DM vs. 206 mg/dL (±46.2 SD) in only DM, with *p* < 0.01 (Figure 1c). Basal triglycerides were 284.5 (±259.1 SD) mg/dL in only DM vs. 106.6 (±55.6 SD) in only TB, with *p* < 0.01, vs. 91.7 (±26.3 SD) mg/dL in only TB post-treatment, with *p* < 0.01. 

### 3.2. Cellular Immunity Evaluation at Baseline: TST and QFT-Plus

The TST was positive in 15/15 (100%) patients with TB-DM and 16/17 (94.1%) with only TB, and it was negative in 0/15 (0%) patients with only DM and 4/15 (26.7%) healthy subjects. QFT-Plus was positive in 14/15 (93.3%) patients with TB-DM, 14/17 (82.4%) patients with only TB, 6/15 (40%) patients with only DM, and 6/15 (40%) healthy subjects. A significant correlation between the TST and QFT-Plus was present in all groups (Kappa > 0.8), except in the DM-only group; there was a correlation between TST and QFT-Plus (Kappa 0.438), with *p* < 0.001 (Table 1).

### 3.3. CD4+/CD8+ Basal and Post Treatment Assessment

**Figure 2:**
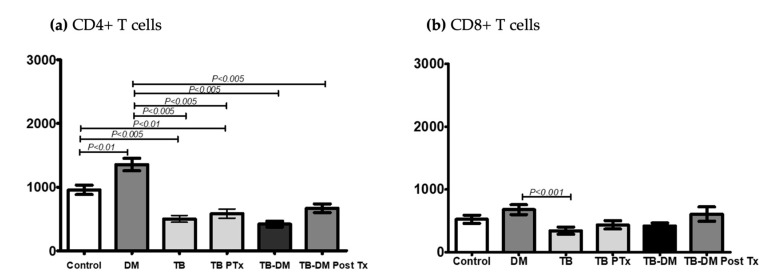
Plots showing the absolute counts of CD4+ and CD8+ T cells in controls (*n* = 15) and patient groups: DM (*n* = 15), TB (*n* = 17), TBPTx (*n* = 12), TB-DM (*n* = 15), and TB-DM PostTx (*n* = 7). (**a**) The absolute counts of CD4+ T cells (cells/μL); (**b**) the absolute counts of CD8+ T cells (cells/μL). ANOVA and Tukey’s multiple comparison were used in both CD4+/CD8+ analyses. The values are expressed as mean ± SD.

In a comparative analysis at baseline, the absolute counts of CD4+ T cells were as follows: 1353 (±377) cells/μL in only DM vs. 956 (±288.3) cells/μL in controls, *p* < 0.01; 1353 (±377) cells/μL in only DM vs. 501.7 (216.4) cells/μL in only TB, *p* < 0.005; and, finally, 1353 (±377) cells/ul in only DM vs. 418.5 (±178.2) cells/μL in TB-DM, *p* < 0.005 (Figure 2a).

After 2 months of anti-TB treatment, the absolute count of CD4+ T cells was 668 (±178.2) cells/μL in the TB-DM group vs. 1353 (±377) cells/μL in only DM, with *p* < 0.005. CD4+ T cells of 501.7 (±216.4) cells/μL in only TB vs. 956 (±288.3) cells/μL in controls, with *p* < 0.01 (Figure 2a).

We also identified a significant difference in the absolute count of CD8+ T cells between the DM-only group with 678 (±299.4) cells/μL and the TB-only group with 343 (±232.2) cells/μL, where *p* < 0.001 (Figure 2b).

### 3.4. Evaluation of Antibodies Immunity

**Figure 3:**
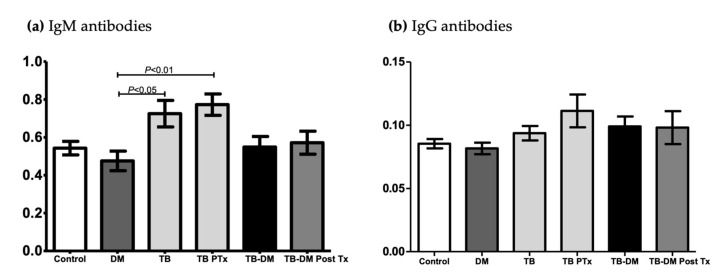
Plots showing the levels of antituberculosis antibodies in controls (*n* = 15) and patients groups: DM (*n* = 15), TB (*n* = 17), TBPTx (*n* = 12), TB-DM (*n* = 15), and TB-DM PostTx (*n* = 7). (**a**) IgM anti-MTB antibodies (absorbance at 490 nm) were analyzed using ANOVA and Tukey’s multiple comparison tests. (**b**) IgG anti-MTB antibodies (absorbance at 490 nm) were analyzed using Kruskal–Wallis and Dunn’s multiple tests. The values are expressed as mean ± SD.

The level of anti-MTB IgM antibodies at baseline was 0.47 (±0.20) in the DM-only group vs. 0.72 (±0.27) in only TB, with *p* < 0.05. There was a significant difference at 2 months of anti-TB treatment between the DM-only group, 0.47 (±0.20), and the TB-only group, 0.77 (±0.20), *p* < 0.01. (Figure 3a) No significant difference was found in anti-MTB IgG antibodies (Figure 3b).

## 4. Discussion

In this study, we compared the immune profiles in patients with pulmonary TB-DM vs. those with only pulmonary TB, only DM, and healthy subjects. We observed differences in cellular and humoral immunity patterns at the pre-treatment stage that were partially corrected after 2 months of treatment. In addition, basal assessment of cellular immunity was abnormal in only the DM group. 

CD4+ T cells play a major role in adaptive immunity against TB, according to our analysis of lymphocytic subpopulations. We found that CD4+ levels were lower in the TB-DM group compared with the other three groups, and there was no recovery after 2 months of anti-TB treatment (only TB and TB-DM groups). This finding is similar to other studies that showed a preponderant role of CD4+ activity in the induction and maintenance of protective immunity against TB through the production of IFN-γ and TNF-α. DM seems to induce a decrease in CD4+ blood levels and its activity in patients with TB, the same as our study [8,9].

Interestingly, according to our results, the persistence of low post-treatment CD4+ levels in active pulmonary TB groups (only TB, TB-DM) was significantly lower than only the DM group and healthy subjects, indicating an absence of cellular immunity recovery after 2 months of treatment. In addition, we compared our low CD4+ levels in active TB with historical controls of other studies and obtained the same results [20]. However, we did not find a significant difference in CD4+ levels within the same TB-DM group (pre- and post-treatment), but their blood value is relevant and could be associated with impaired immune recovery, according to other published studies. In our study, subjects presented with low CD4+ levels at baseline and persisted after 2 months of anti-TB treatment, suggesting an impaired immune recovery [16].

Regarding the CD8+ T cell analysis, we identified higher blood values in the DM-only group compared with the TB-only group, which may be explained by the high prevalence of LTBI in patients with DM, similar to other studies that found that the frequency of antigen-specific CD8+ T cells was higher in individuals with LTBI and could be associated with a protective effect to avoid the progression to active TB [12].

In our study, IgM levels were elevated in patients with only TB at baseline, which indicates active TB. However, 2 months post-anti-TB treatment, levels remained significantly elevated in the same group. We do not have a clear explanation for this paradoxical response. However, it could be a result from a slower decrease in blood levels, which could return to normal in a subsequent analysis after 3 or 4 months of treatment. In addition, there is no difference in IgM and IgG in patients with TB-DM [14].

Finally, the TST and QFT-Plus exhibited good agreement regarding active TB (TB-only and TB-DM groups), but the TST performed poorly in the DM-only group compared to QFT-Plus, which may reflect anergy due to impaired immunity in this population that leads to false negative results of the TST. These results suggest that QFT-Plus should be used instead of the TST when LTBI is suspected in patients with DM, as per the WHO’s recommendations [4]. 

There have been hypotheses on cellular immunity behavior in patients with TB, which we identified in our research. A study demonstrated an alteration in the transcriptomes of patients with both TB and DM, which reduced type I immunity and interferon responses in correlation with intermediate and elevated glycosylated hemoglobin levels [21].

Other studies have associated lower CD4+ T cell and IL-10 activity in the blood and lungs with elevated glycosylated hemoglobin levels that persisted after anti-TB treatment [22]. Hypertriglyceridemia associated with foamy macrophages and low levels of HDL cholesterol has been associated with tissue damage [23]. Nonetheless, we did not identify a significant difference in triglyceride blood levels. Still, we found low levels of HDL cholesterol that recovered after anti-TB treatment in the TB-DM group, as reported in other studies [24].

Limitations to this study include a small sample size and loss to follow-up. It is also a single-center study. We did not measure phagocytosis, other lymphocyte subpopulations, or cytokines, and we did not evaluate the anti-DM treatment associated with immunity. 

Other immunity function alterations in elevated glycosylated hemoglobin levels involve monocyte activation, antigen presentation, and phagocytosis [23], which we, unfortunately, did not evaluate. We focused only on the baseline and after two months of intensive anti-TB treatment. We did not follow up until the completion of treatment after 6 or 9 months, which could be an interesting evaluation for future studies.

## 5. Conclusions

There is an alteration of adaptive immunity CD4+ T cells in patients with TB and DM which does not recover after 2 months of anti-TB treatment. We need more studies to confirm this finding.

## Figures and Tables

**Table 1:** Demographics and patient characteristics *.

Variables	TB-DM (*n* = 15)	Only TB (*n* = 17)	Healthy Subjects (*n* = 15)	Only DM (*n* = 15)
Sex Female, n (%) Male, n (%)	- 4 (26.7) 11 (73.3)	- 5 (29.4) 12 (70.6)	- 8 (53.3) 7 (46.7)	- 6 (40) 9 (60)
Age, median (range)	46 (22–71)	27 (18–84)	36 (21–70)	57 (47–75)
BMI, median (range)	17.2 (14.6–19.5)	15.88 (13–22)	18.3 (12–23)	17.2 (14.8–22.3)
Positive TST, n (%)	15 (100%)	16 (94.1%)	4 (26.7%)	0 (0%)
QFT–Plus Positive, n (%) Negative, n (%) Indeterminate, n (%)	- 14 (93.3%) 0 (0%) 1 (6.7%)	- 14 (82.4%) 2 (11.8%) 1 (5.9%)	- 6 (40%) 9 (60%) 0 (0%)	- 6 (40%) 6 (40%) 2 (20%)

* Refer to Figure 1 for baseline and follow-up results.

## Data Availability

The data presented in this study are available on request from the corresponding author. The data are not publicly available due to privacy restrictions.
